# Beyond the Operating Room: The Lasting Impact of Preoptimization in Abdominal Wall Reconstruction

**DOI:** 10.1097/AS9.0000000000000648

**Published:** 2026-01-12

**Authors:** Victoria L. Walker, Lucy R. Hinton, Samantha W. Kerr, Alexis M. Holland, Vedra A. Augenstein, B. Todd Heniford, Sullivan A. Ayuso

**Affiliations:** From the * Department of Surgery, Baptist Health Sciences University, Memphis, TN; † Division of Gastrointestinal and General Surgery, Department of Surgery, Endeavor Health, Evanston, IL; ‡ Division of Gastrointestinal and Minimally Invasive Surgery, Department of Surgery, Carolinas Medical Center, Charlotte, NC; § Department of Plastic and Reconstructive Surgery, Ohio State University Medical Center, Columbus, OH; ‖ Division of Elective General Surgery, Department of Surgery and Perioperative Care, University of Texas at Austin, Dell Medical School, Austin, TX.

**Keywords:** abdominal wall reconstruction, comorbidity management, long-term benefits, preoptimization

Prehabilitation in abdominal wall reconstruction (AWR) represents an active, structured approach to preparing patients for surgery, encompassing interventions such as smoking cessation, weight reduction, comorbidity management, physical conditioning, and coordinated multidisciplinary care. These preparatory measures are designed to improve physiologic reserve, mitigate perioperative risk, and set the stage for a more robust postoperative recovery. In our 2024 *Annals of Surgery Open* perspective, “The Role of Prehabilitation in AWR: It’s More than Watch and Wait,” we acknowledged that while prehabilitation demonstrably improves short-term and intermediate outcomes, it demands significant commitment, organizational infrastructure, and substantial engagement from the surgeon.^1^ However, a central and unanswered question persists: do these improvements confer benefits that endure over the years, or are they transient gains that diminish once structured support ends? And if they do endure, what are the specific benefits that this may entail?

Since the publication of our previous surgical perspective piece in Annals, we have attempted to address prehabilitation sustainability for AWR patients. Our structured, supervised prehabilitation protocol sets individualized weight loss, glycemic, dietary, and smoking cessation targets, with regular monitoring, ancillary referrals (eg, endocrinology and bariatrics) for poor progress, and follow-up in office visits.^2^ All patients are counseled on aerobic exercise, adherence to a ketogenic diet, glycemic control (HbA1c ≤7.2%), and smoking cessation for ≥4 weeks. Holland et al^2^ reviewed our prospectively maintained institutional database for patients undergoing open AWR and examined the effects of our structured, supervised prehabilitation program. On average, patients lost 26 pounds over 10 months and sustained a mean loss of 24 pounds over 3.5 years of follow-up after surgery. Notably, nearly 50% continued to lose weight beyond the perioperative period, losing an average of more than 20 additional pounds. This finding demonstrates that preoperative weight loss is not only a temporary sacrifice for surgical eligibility but also the catalyst needed for a broader lifestyle change.

In a subsequent study by Holland et al^3^, preoptimization was evaluated in diabetic patients and active smokers who were scheduled for elective AWR. These patients were counseled before surgery, during which specific prerequisites were set out—a glycated hemoglobin (Hgb A1c) of ≤7.2% and at least 4 weeks of abstinence from smoking. More than 3 years after surgery, 63% maintained a Hgb A1c of ≤7.2% and 96% of those patients showed additional HbA1c reduction. Similarly, nearly 60% of smokers remained abstinent long-term. Such behavioral changes further reinforce that surgeon-directed optimization protocols may be a “teaching moment” for lasting health behavior modification.

Although not hernia specific, our findings are supported by previous work by Sadr Azodi et al,^4^ who published a randomized controlled trial (RCT) demonstrating the sustained impact of successful smoking cessation programs in a perioperative setting for elective general and orthopedic surgical procedures.^4^ Participants who received weekly counseling and free nicotine replacement therapy had a 36% perioperative abstinence rate compared with 2% in the control group. Sustained abstinence at 1 year further demonstrated the intervention’s durability. Furthermore, Fong et al’s^5^ systematic review and meta-analysis of RCTs confirmed that smoking cessation is associated with significantly improved likelihood of being abstinent from smoking 1 year following surgery.^5^

## WHY DOES IT MATTER?

The short-term benefits of prehabilitation, namely a reduction in surgical site infection and a resultant decreased incidence of hernia recurrence, are well-documented and grounded in basic physiology.^6^ The long-term benefits of these behaviors, however, appear even more meaningful. Obesity continues to increase the likelihood of hernia recurrence in the long term and is the main driver of metabolic syndrome, which has a multisystemic impact that affects cardiovascular function, insulin sensitivity, increases the risk of cancer, liver dysfunction, etc.^7^ Smoking accounts for 11% of all of the world’s deaths and over a quarter of ischemic heart disease-related mortalities, while poor glycemic control can lead to renal and eye disease and worsening cardiovascular health. Undeniably, optimizing each of these factors—smoking cessation, weight loss, and improved glycemic control—can extend one’s lifespan by over a decade and reduce morbidity.^8,9^

In addition to extending the quantity of one’s life, there are financial implications to the success of prehabilitation efforts that should be considered. From a hernia perspective, data generated from models like CeDAR shows that a reduction in a single postoperative infectious complication can lead to tens of thousands of dollars in cost savings.^10^ These financial estimations do not even account for the need for reoperations and healthcare expenses that result from complications that lead to hernia failure. Meanwhile, the sustained improvements in overall health have a downstream financial impact that likely far surpasses hernia-related complications. Decreased healthcare utilization from comorbidity improvements and the reduction of the need for expensive chronic treatments, such as insulin or dialysis, will drastically drive down healthcare costs.

There is predictably a selection bias for those patients who are successfully optimized before surgery. These patients have demonstrated a commitment to making important health-related changes before their operation, making it easier to maintain these habits for patients who were unable to have the initial buy-in. We recognize that we did not present, nor do we have data for patients for whom prehabilitation efforts were recommended but did not follow through. However, based on our anecdotal experience, this is exceedingly low once patients realize that these efforts will help them achieve the best possible results. A further limitation is multiple confounding variables complicating assessment of prehabilitation’s durable postoperative impact, and it is possible that the surgery itself made it easier for patients to maintain these health efforts, and may be an added benefit to the operation. Regardless, the impending necessity of surgery provides a potent motivational framework rarely replicated in other clinical contexts. The adherence observed in these patient populations suggests that the surgeon-patient relationship, particularly when framed around preoperative optimization, fosters a sense of accountability and trust that facilitates sustained lifestyle modification.

The overarching message is that contemporary advances in perioperative optimization not only improve surgical outcomes but can extend longevity and enhance the quality of life for surgical patients. Historically, the metrics by which surgeons have gauged success have been anchored in technical endpoints such as fascial closure rates, incidence of wound complications, and hernia recurrence, providing an essential but incomplete picture of surgical value. While these measures remain indispensable to operative quality assurance, they fail to capture the broader trajectory of patient health once the immediate postoperative period has passed. Prehabilitation protocols should be reframed not as discrete perioperative interventions, but as shared, longitudinal goals supported by structured follow-up extending well beyond the traditional global period. Such an approach positions the surgeon not merely as an operator but as a central architect of durable health benefits.

Taken together, these recently published studies underscore an expanded professional responsibility for the modern surgeon, one that transcends operative technical proficiency to encompass active engagement in long-term patient health optimization. In our practice, the conversation about a patient’s weight and other comorbidities, such as smoking and diabetes, does not stop at the preoperative visit. Rather, this is part of the continuing conversation that is had at the first postoperative visit and subsequent surveillance visits. We routinely follow patients out to a year after their hernia repair and inquire about these specific behaviors at each time point. Even if we are not able to make medication changes or follow lifestyle interventions in an ongoing fashion, we are intentional about communicating with patient’s primary care providers and subspecialists to be a part of a coordinated effort to improve a patient’s overall health. Multidisciplinary collaboration remains the cornerstone of best practice in healthcare efficiency and efficacy.

A focus on prehabilitation constitutes a paradigm shift in surgical philosophy, redirecting focus from complication avoidance alone toward proactive improvement of physiologic reserve and overall wellness. Evidence increasingly supports that patients undergoing targeted prehabilitation enter surgery with improved baseline status, experience fewer perioperative setbacks, and recover more rapidly with subsequent benefits that persist for years. By explicitly embracing optimization as a core criterion for surgical success, we elevate the role of the surgeon from procedural technician to longitudinal health steward. Ultimately, the integration of prehabilitation and long-term optimization into surgical care represents a practical evolution of the profession, ensuring that the impact of our interventions is measured not only in operative success but also in the number of years and vitality they help preserve.

Our hernia community should embrace prehabilitation and reaffirm its place in AWR practice. Rather than challenge its effectiveness, the community should recognize the need for greater investment in supportive systems and resources that enable all patients to participate fully in their care. Telehealth check-ins, social work partnerships, and tailored education materials can help bridge these gaps. Additionally, we should look for other ways to optimize patients, such as targeting frailty through preoperative physical therapy programs and partnerships with geriatric medicine, which can expand the repertoire of preoperative services that can impact patient care. As we continue to emphasize improvement in quality of life as hernia surgeons, we should feel confident and inspired that we have the ability to do so much more.
